# Pregnancy‐associated venous insufficiency course with placental and systemic oxidative stress

**DOI:** 10.1111/jcmm.15077

**Published:** 2020-03-06

**Authors:** Miguel A. Ortega, Beatriz Romero, Ángel Asúnsolo, Clara Martínez‐Vivero, Felipe Sainz, Coral Bravo, Juan De León‐Luis, Melchor Álvarez‐Mon, Julia Buján, Natalio García‐Honduvilla

**Affiliations:** ^1^ Department of Medicine and Medical Specialties Faculty of Medicine and Health Sciences University of Alcalá Alcalá de Henares Spain; ^2^ Networking Biomedical Research Center on Bioengineering, Biomaterials and Nanomedicine (CIBER‐BBN) Ramón y Cajal Institute of Sanitary Research (IRYCIS) Alcalá de Henares Spain; ^3^ Department of Surgery, Medical and Social Sciences Faculty of Medicine and Health Sciences University of Alcalá Alcalá de Henares Spain; ^4^ Angiology and Vascular Surgery Unit Central University Hospital of Defense‐UAH Madrid Spain; ^5^ Service of Gynecology and Obstetrics Central University Hospital of Defense‐UAH Madrid Spain; ^6^ Service of Gynecology and Obstetrics Section of Fetal Maternal Medicine University Hospital Gregorio Marañón Madrid Spain; ^7^ Immune System Diseases-Rheumatology and Oncology Service University Hospital Príncipe de Asturias, CIBEREHD Alcalá de Henares Spain

**Keywords:** ERK, inducible nitric oxide synthase, Lower extremity venous insufficiency, malondialdehyde, NOX1, NOX2, PARP, Pregnancy

## Abstract

The development of lower extremity venous insufficiency (VI) during pregnancy has been associated with placental damage. VI is associated with increased oxidative stress in venous wall. We have investigated potential disturbance/dysregulation of the production of reactive oxygen species (ROS) in placenta and its eventual systemic effects through the measurement of malondialdehyde (MDA) plasma levels in women with VI. A total of 62 women with VI and 52 healthy controls (HCs) were studied. Levels of nicotinamide adenine dinucleotide phosphate‐oxidase 1 (NOX1), 2 (NOX2), inducible nitric oxide synthase (iNOS), endothelial (eNOS), poly(ADP‐ribose) polymerase PARP (PARP) and ERK were measured in placental tissue with immunohistochemistry and RT‐qPCR. Plasma and placental levels of MDA were determined by colorimetry at the two study times of 32 weeks of gestation and post‐partum. Protein and gene expression levels of NOX1, NOX2, iNOS, PARP and ERK were significantly increased in placentas of VI. eNOS activity was low in both study groups, and there were no significant differences in gene or protein expression levels. Women with VI showed a significant elevation of plasma MDA levels at 32 weeks of gestation, and these levels remained elevated at 32 weeks post‐partum. The MDA levels were significantly higher in placentas of women with VI. Placental damage that was found in the women with VI was characterized by overexpression of oxidative stress markers NOX1, NOX2, and iNOS, as well as PARP and ERK. Pregnant women with VI showed systemic increases in oxidative stress markers such as plasma MDA levels. The foetuses of women with VI had a significant decrease in their venous pH as compared to those from HC women. The situation of oxidative stress and cellular damage created in the placenta is in coexpression with the production of a pH acidification.

## 1 INTRODUCTION

Lower extremity venous insufficiency (VI) is a vascular disorder that is characterized by alteration of the peripheral venous system that reduces or impedes venous return.^1^, ^2^ Various epidemiological studies worldwide provide evidence that VI is one of the chronic diseases that shows the greatest variability in its incidence and prevalence.^3^ The yearly incidence of varicose veins in women is 2.6%, according to the Framingham study.^4^ Pregnancy is one of the factors that increases the risk of VI development.^5^ Consequently, VI is a common complication of pregnancy and affects a third of women during their first pregnancy.^6^, ^7^ Several pathogenic mechanisms appear to be involved in this association during pregnancy, including an increased abdominal pressure and blood volume and an enhanced cardiac output with a secondary augmentation of the venous pressure in the lower extremities.^8^, ^9^ Furthermore, the hormonal changes experienced by pregnant women may also contribute to venous damage.^10^ Age, obesity, sex hormones and family history of VI increase the risk of varicose veins during pregnancy.^7^, ^10^, ^11^, ^12^

Interestingly, pregnancy‐associated VI appears to involve more venous territories than those that are clinically evident in the lower extremities. An unexpected association of VI with the involvement and damage of the placenta has been recently shown.^9^ The placental tissues of women with pregnancy‐associated VI show structural remodelling and hypoxic cellular stress with a higher number of villi and syncytial knots and enhanced apoptotic cellular death.^9^, ^13^

The formation of the placental lesions is driven by the compromised arterial blood flow, but its pathogenesis is complex, and several molecular pathways appear to be involved. One of these pathways is the overexpression of different enzymes that produce the components of reactive oxygen species (ROS) in the villi of the placenta, which can be damaged by arterial diseases such as pre‐eclampsia and arterial hypertension.^14^, ^15^, ^16^ Furthermore, ROS overexpression is found in the venous wall of VI patients.^17^ An imbalance between ROS production and antioxidant defence mechanisms results in an oxidative stress that can produce different molecular alteration phenomena such as the oxidation of lipids, proteins and nucleic acids, enzyme inactivation and tissue inflammation.^18^, ^19^, ^20^ Furthermore, the interaction of ROS with nitric oxide (NO) promotes its impact on the cellular ageing process.^21^, ^22^ NO is considered to be a critical mediator of the cellular damage that is induced by oxidative stress.^22^, ^23^ The induced isoform (iNOS) and endothelial nitric oxide synthase (eNOS) are the main sources of cellular NO.^19^, ^24^ Disturbances in iNOS and eNOS have been described in the placentas of patients with pre‐eclampsia.^25^, ^26^ Cellular oxidative stress is also linked to the increased generation of ROS through the activation of the transmembrane nicotinamide adenine dinucleotide phosphate‐oxidase (NOX) enzyme family.^27^ The isoforms NOX1 and NOX2 are mayor players in the production of superoxide anion.^19^ It has been shown that both isoforms have increased expression in the placentas of women with vascular hypertension such as pre‐eclampsia, showing an alteration of its expression and activity in these vascular pathologies.^28^, ^29^ Authors have observed how oxidative stress and metabolic changes can create DNA damage that is associated with an increase in poly(ADP‐ribose) polymerase PARP (PARP) repair activity.^30^, ^31^, ^32^ In this sense, the mitogen‐activated protein kinase/extracellular signal‐regulated (MAPK/ERK) pathway has been mentioned as a point of great importance in cellular damage processes. Signalling pathways (MAPK/ERK) play fundamental roles in a wide range of cellular processes and are often deregulated in disease states. An important mode of action for these pathways is to control gene expression, in particular by regulating transcription with great tissue impact.^33^, ^34^

One of the points that is pointed out in an important way to know the damage that oxidative stress causes in cells is the production of malondialdehyde (MDA) as a result of lipid peroxidation. The particular reaction of ROS with lipids is generally known as ‘lipid peroxidation’. Isoprostane MDA is a widely accepted biomarker of oxidative stress with biological consequences.^35^

It is possible that the placental hypoxaemic damage that is found in women with pregnancy‐associated VI might be associated with an abnormal ROS regulation in the placental tissue. This potential oxidative stress in the placenta might also have systemic effects such as increased levels of oxidative metabolites. In this work, we investigated the expression levels of the iNOS, eNOS, NOX1, NOX2 and PARP enzymes in the different cellular components of the placenta from women with pregnancy‐associated VI. We studied the placentas of women with pregnancy‐associated VI in parallel with the healthy control (HC) group. In addition, we analysed the placental tissue and peripheral blood MDA levels as biomarkers of the oxidative stress and physiological stress that occurs in women with pregnancy‐associated VI and the HCs, both during pregnancy and after delivery.

## 2 MATERIALS AND METHODS

### 2.1 Study design

In this prospective work, we studied 62 women who were diagnosed as having VI without a concomitant disease during 32 weeks of their pregnancy (women with pregnancy‐associated VI). The study point at 32 weeks of gestational age is an international consensus standard in the study of placental insufficiency. The 32‐week study is due to foetal maturity and other maternal‐foetal structures.^36^, ^37^ Women in the VI were newly diagnosed without and previous clinical evidence of the disease. In parallel, we studied 52 women who were healthy for the same 32 weeks of pregnancy, and we also studied them throughout their remaining pregnancy (HC).

This study was conducted in women that visited the obstetric consultations in the 32 weeks of gestation. Once they signed the informed consent form, a clinical history, general physical examination and laboratory measurements were obtained, and an exploration of their lower limbs was performed with an Eco‐Doppler (portable M‐Turbo Eco‐Doppler; SonoSite, Inc.) transducer at 7.5 MHz. The examination was performed, while the women were in a standing position and the leg was explored in external rotation and with support in the contralateral leg. The study included the major saphenous axis from the inguinal region to the ankle and femoral veins. The classification of VI in the pregnant women under study was performed according to the Classification System for Chronic Venous Disorders (CEAP).^38^ The pregnancy was routinely monitored at the centre, and placental samples were obtained from the women at their time of delivery.

*Inclusion criteria* were women between 18 and 39 years of age in the 32 weeks of pregnancy with clinical evidence of VI in the lower limbs with a CEAP classification of C1 or higher. *Exclusion criteria* included the following: 1. previous diabetes mellitus and endocrinology diseases; 2. previous blood hypertension; 3. systemic and organ‐specific autoimmune disease; 4. active infectious diseases and chorioamnionitis; 5. venous malformations; 6. heart, kidney and lung insufficiencies; 7. pre‐eclampsia and/or Hellp syndrome; 8. known causes of intrauterine growth restrictions; 9. body mass index (BMI) ≥ 25 kg/m^2^; 10. unhealthy habits (eg alcohol, tobacco consumption); 11. presence of pathologic lesions such as villous infarcts, avascular villi, delayed villous maturation, chorangiosis or chronic villitis; 12. appearance of any of the previous exclusion criteria during the 9 months of follow‐up; and 13. previous evidence of VI. In the HC group, we included age‐matched pregnant women without clinical evidence of VI and without the exclusion criteria. The present study was conducted in accordance with respect for the basic ethical principles autonomy, beneficence, non‐maleficence and distributive justice, and its development followed the rules of Good Clinical Practice, the principles enunciated in the last Declaration of Helsinki (2013) and the Oviedo Convention (1997). The patients were duly informed, and each was asked to provide written informed consent. The project was approved by the Ethics Committee of Clinical Investigations of the University Central Hospital of Defense Gómez‐Ulla‐UAH (37/17).

### 2.2 Samples

The placental tissue biopsies were taken once the placenta was expelled and used to immunohistochemical, genetic and molecular studies. In all cases, five fragments of the placenta were taken with a scalpel to ensure that the samples included several cotyledons (placentons). These fragments were introduced into two different sterile tubes: one containing minimum essential medium (MEM) with 1% antibiotic/antimycotic (both from Thermo Fisher Scientific) and another containing RNAlater^®^ Solution (Ambion). In the laboratory, the samples were processed in a laminar flow bell class II Telstar AV 30/70 Müller 220 V 50 MHz (Telstar SA Group), thus allowing an environment of sterility. The preserved samples were kept in 1 mL of RNAlater^®^ at −80°C until further processing for analysis of gene expression. The samples that were conserved in MEM were reserved for histological and immunodetection studies.

Blood samples were obtained from the study population by puncturing the superficial vein of the elbow fold after placing a compressor on the arm (the extraction was done in the morning and fasting for 10 hours). Plasma was obtained from said blood samples for MDA study. The entire volume of the blood sample was transferred from the heparin tubes to the sterile centrifuge tubes, where they were centrifuged at 1500 *g* for 15 minutes. Next, the plasma was collected and transferred to 1.5‐mL Eppendorf tubes, where they were kept frozen at −80°C until they were used for the study. The haematological and biochemical parameters were analysed by the Clinical Analysis Service of the University Hospital according to standardized protocols.

### 2.3 Genetic or molecular studies

The amounts of cDNA in each sample were quantified with real‐time polymerase chain reaction (qPCR) of the following genes of interest: NOX1, NOX2, iNOS, eNOS and PARP. The results were normalized using the constitutively expressed gene TBP (TATA box‐binding protein). De novo primers or primers that were specific for all genes of interest were designed (Table S1) using the online applications Primer‐BLAST^39^ and AutoDimer.^40^ RNA was extracted using the guanidine‐phenol‐chloroform isothiocyanate method described by Ortega et al.^41^

The qPCR was performed in a StepOnePlus™ System (Applied Biosystems—Life Technologies) using the relative standard curve method. The reaction was performed as follows: 5 µl of each sample containing cDNA diluted 1/20 in nuclease‐free water was mixed with 10 µL of iQ™ SYBR^®^ Green Supermix (Bio‐Rad Laboratories), 1 µL of forward primer (6 μmol/L), 1 µL of reverse primer (6 μmol/L) and 3 µL of DNase‐ and RNase‐free water in a MicroAmp^®^ 96‐well plate (Applied Biosystems—Life Technologies) for a total reaction volume of 20 µL.

### 2.4 Immunohistochemical studies

The samples that were preserved in MEM were washed/hydrated several times with medium without antibiotic to remove blood cells and were cut into fragments that were kept in different fixatives, including F13 (60% ethanol, 20% methanol, 7% polyethylene glycol and 13% distilled H_2_O). After the samples were fixed for the necessary amount of time in each fixing solution, they were dehydrated following standardized protocols.^42^

The antigen‐antibody reaction was detected with the ABC (avidin‐biotin complex) method with peroxidase or alkaline phosphatase as a chromogen, according to the following protocol: 1. wash the samples three times with 1× PBS 1× for 5 minutes each; 2. block the non‐specific binding sites with 3% bovine serum albumin (BSA) in PBS for 30 minutes at room temperature; 3. incubate with the primary antibody (Table S2) diluted in 3% BSA and PBS overnight at 4°C; 4. rinse three times with PBS for 5 minutes each; 5. incubate with the secondary antibody bound to biotin (rabbit IgG, diluted 1/300 [RG‐96; Sigma‐Aldrich], goat IgG with 1/100 dilution [GT‐34/ B3148; Sigma‐Aldrich] and mouse IgG with 1/300 diluted [F2012/045K6072; Sigma‐Aldrich]) and diluted in PBS for 1 hour and 30 minutes at room temperature; 6. rinse three times with PBS for 5 minutes each; 7. incubate with the avidin‐peroxidase conjugate ExtrAvidin^®^‐Peroxidase (Sigma‐Aldrich) for 60 minutes at room temperature (diluted 1/200 in PBS); 8. rinse three times in PBS for 5 minutes each; 9. reveal by incubation with the chromogenic substrate diaminobenzidine (Kit DAB, SK‐4100; Vector Laboratories); the chromogenic substrate was prepared immediately before exposure (5 mL of distilled water, two drops of buffer, four drops of DAB, two drops of hydrogen peroxide); this technique allows for brown‐coloured staining; 10. rinse three times with distilled water for 5 minutes each to stop the development of the reaction; 11. stain the nuclei, for contrast, with Carazzi's haematoxylin for 5‐15 minutes; 12. rinse in running water for 10 minutes; and 13. mount in aqueous medium with plasdone. In all of the immunohistochemical studies, sections of the same tissue were used as a negative control in which the incubation with the primary antibody was replaced by an incubation in the blocking solution.

For each of the patients in the established groups, 5 sections and 10 fields per section were examined by random selection in placental villi and decidual cells. The patients were described as positive when the average marked area in the analysed sample was greater than or equal to 5% of the total, following the anatomopathological protocol of Cristobal et al.^42^ Positive cells for immunohistochemical studies were counted under a microscope (1000×) in 10 random areas (0.5 mm^2^ per patient). The preparations were examined under a Zeiss Axiophot optical microscope (Carl Zeiss) that was equipped with an AxioCam HRc digital camera (Carl Zeiss).

### 2.5 Lipid peroxidation assay

A colorimetric lipid peroxidation assay kit (ab118970; Abcam) is a convenient tool for the sensitive detection of MDA in a variety of samples. The MDA that is present in the sample reacts with thiobarbituric acid (TBA) to generate an MDA‐TBA adduct that is quantified colorimetrically (OD 532 nm). This assay detects MDA levels as low as 0.1 mol/well of MDA (1 mol/well of MDA in colorimetry). The present study quantified the MDA levels in the placental tissue samples and the blood plasma of the pregnant woman at 32 weeks of gestation and at 32 weeks post‐partum. To conduct the experiment, the following protocol was used: 1‐3. MDA lysis buffer, phosphotungstic acid solution and butylated hydroxytoluene (BHT; 100X): ready for use as supplied; the buffer was stored at −20°C and brought to ambient temperature before use; 4. TBA solution: a vial of TBA was reconstituted in 7.5 mL of glacial acetic acid, where it then was transferred to another tube and the final volume was adjusted to 25 mL with ddH2O; the sample was mixed well to dissolve, sonication was performed in an RT water bath, and the sample was stored at 4°C; and 5. standard MDA (4.17 mol/L): ready for use as supplied; the solution was stored at −20°C and brought to ambient temperature before use.

#### 2.5.1 Tissue samples

A total of 20 mg of placental tissue was used for the analysis. The protocol that was used was as follows: 1. rinse the tissue in cold PBS; 2. prepare the lysis solution: 300 mL of lysis buffer with MDA and 3 µL of BHT (100X); 4. homogenize the tissue in 303 µL of lysis solution (buffer + BHT) with 10‐15 passes with a homogenizer on ice; and 5. centrifuge at 13 000 *g* for 10 minutes to remove the insoluble material and collect the supernatant.

#### 2.5.2 Plasma samples

Quantities of 10 µL of blood plasma were used from the two study times (32 weeks of gestation and 32 weeks post‐partum). The protocol that was used was as follows: 1. carefully mix 10 µL of plasma with 500 µL of 42 mmol/L H2SO4 in a microcentrifuge tube; 2. add 125 µL of phosphotungstic acid solution and mix by vortexing; 3. incubate at room temperature for 5 minutes; 4. centrifuge at 13 000 *g* for 3 minutes; 5. collect the precipitate and resuspended on ice with 100 µL of ddH2O (with 2 µL of BHT [100X]); and 6. adjust the final volume to 200 µL with ddH2O.

For the colorimetric assay, a 0.1‐mol/L MDA standard is prepared by diluting 10 µL of 4.17 mol/L MDA standard in 407 µL of ddH2O. A 2‐mmol/L MDA standard was made by diluting 10 µL of 0.1 mol/L MDA standard in 490 µL of ddH2O. The MDA standard (2 mmol/L) was used, and dilutions were prepared for the standard curve. The MDA‐TBA adduct was generated by the addition of 600 µL of TBA reagent in each vial. The samples were then incubated at 95°C for 60 minutes. Each sample was cooled to room temperature in an ice bath for 10 minutes. Then, 200 µL of the TBA/standard mixture (labelled as standard) and 200 µL of the TBA/sample mixture (labelled as sample) were combined in a 96‐well microplate for analysis. For greater sensitivity, 300 µL of n‐butanol was added to precipitate the MDA‐TBA adduct. If there was no separation, 100 µL of 5 mol/L NaCl was added to the mixture and then vigorously stirred. The layers could then be separated by centrifugation (3 minutes at 16 000 *g*). The MDA‐TBA adduct phase was transferred to a new tube, and the n‐butanol was evaporated. The MDA‐TBA adduct was dissolved in 200 µL of ddH2O and then placed in a 96‐well microplate plate for analysis. The product was measured immediately in a microplate reader at OD 532 nm for the colorimetric assay. The measurement was made with a Victor2 multifunction device (Wallac, Victoria, Australia).

### 2.6 Statistical analysis

For the statistical analysis, the GraphPad Prism^®^ 6.0 program was used, and the Mann‐Whitney *U* test was applied. The data are expressed as the median with interquartile range (IQR). The significance was set at *P* < .05 (*). If the study variables were not quantitative, we used Pearson's chi‐squared test or Fisher's exact test, when applicable.

## 3 RESULTS

### 3.1 Clinical and demographic characteristics

The complete study was performed with 114 patients, including 62 women with VI during pregnancy and 52 control women without evidence of VI during pregnancy. The mean age is 33 [22‐40] VI and 34 [27‐41] HC. Not exist significant differences in the mean age of patients (*P* = .06915). The gestational age (weeks) is 40.5 [39‐41.5] VI and 41 [39‐42] HC. The number of caesarean delivery is 12 (19.4%) in VI and 9 (17.3%) in HC. We did not find significant differences between the VI and HC groups in their gestational age, number of previous pregnancies, previous abortions, regular menstrual cycles, type of profession‐sedentary, placental weight and size (Table 1). Pregnant women with VI had a significantly higher prevalence of family history of VI (n = 42 [67. 7%]) compared to those in the HC (n = 17 [32.7%]) group, OR = 4.32 [1.84‐10.27] (*P* = .0002). According to the CEAP diagnosis, the women with pregnancy‐associated VI were at grade ≥C1, class 1 [n = 37 (59.7%)]: telangiectases or reticular veins; class 2 [n = 21 (33.8%)]: varicose veins; and class 3 (n = 4 [6.5%]: oedema).^38^ We did not observe any pathologic lesions, such as villous infarcts, avascular villi, delayed villous maturation, chorangiosis or chronic villitis.

**Table 1:** Demographics and clinical characteristics of pregnant women with lower extremity venous insufficiency (VI) and pregnant women health control (HC)

	VI (n = 62)	HC (n = 52)
Median age [IQR]	33 [22‐40]	34 [27‐41]
Gestational age (wk) median [IQR]	40.5 [39‐41.5]	41 [39‐42]
C‐section delivery	12 (19.4%)	9 (17.3%)
Varicose vein (CEAP)		
C1	37 (59.7%)	0 (0%)
C2	21 (33.8%)	0 (0%)
C3	4 (6.5%)	0 (0%)
Previous pregnancies	33 (53.2%)	19 (36.5%)
Previous abortions	14 (22.6%)	9 (17.3%)
Family history*	42 (67.7%)	17 (32.7%)
Regular menstrual cycles	50 (80.6%)	42 (80.7%)
Type of profession‐sedentary	41 (66.1%)	40 (76.9%)
Placental weight (gr)	490 [390‐630]	500 [350‐610]
Placental size (cm)	20 [17‐23]	21 [18‐25]

Note

Abbreviations: gr = grams, cm = centimetres.

* *P* = .0002.

### 3.2 Placentas from women with pregnancy‐associated VI show increased NOX‐1 and NOX‐2 gene and protein expression

**Figure 1:**
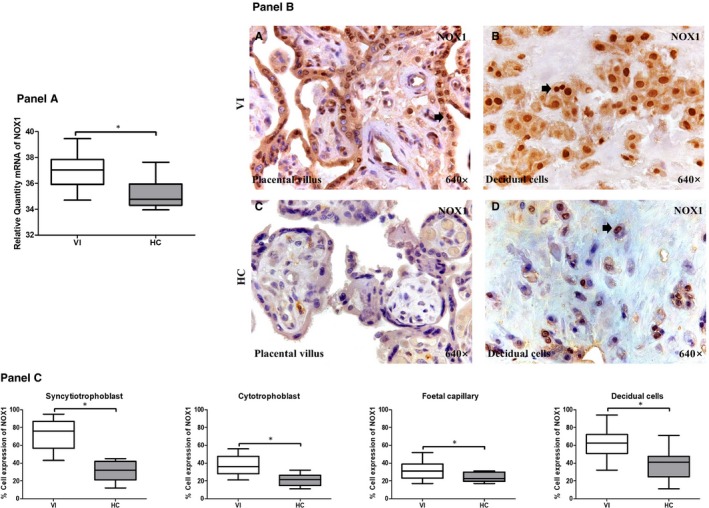
Panel A, NOX1 gene expression between pregnant women with lower extremity venous insufficiency (VI) and pregnant women health control (HC), as measured by RT‐qPCR. Panel B, Histological images of NOX1 protein expression showing the relevant immunodetection in the placental villus (C) and decidual cells (D) in the placentas of women with VI. Panel C, Quantification of the percentages of syncytiotrophoblast, cytotrophoblast, foetal capillary and decidual cells that stain positive for NOX1. The arrows are the brown coloration indicating the specific protein expression, and 640× is the magnification of histological images. Data shown median and IQR. *P‐value < .05 (Mann‐Whitney *U* test)

First, we studied the levels of NOX‐1 and NOX‐2 gene expression by RT‐qPCR in the placentas of women with pregnancy‐associated VI and in the HC group. The NOX‐1 gene expression in the placentas from the women with pregnancy‐associated VI (37.03 [34.70‐39.46] relative quantity mRNA [RQ]) was significantly higher than that found in the placentas from the HC group (34.77 [33.95‐37.63] RQ; *P* = .0002; Figure 1, Panel A).

**Figure 2:**
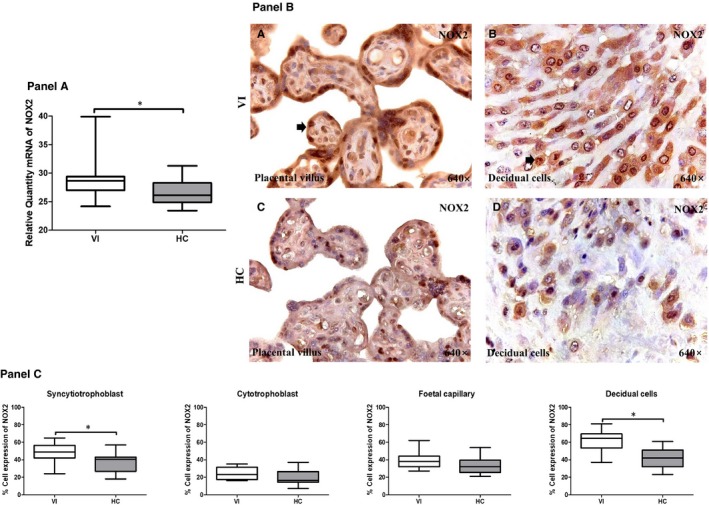
Panel A, NOX2 gene expression between pregnant women with lower extremity venous insufficiency (VI) and pregnant women health control (HC), as measured by RT‐qPCR. Panel B, Histological images of NOX2 protein expression showing the relevant immunodetection in the placental villus (C) and the decidual cells (D) in the placentas of women with VI. Panel C, Quantification of the percentages of syncytiotrophoblast, foetal capillary and decidual cells that stain positive for NOX2. The arrows are the brown coloration indicating the specific protein expression, and 640× is the magnification of histological images. Data shown median and IQR. *P‐value < .05 (Mann‐Whitney *U* test)

We investigated NOX‐1 and NOX‐2 protein expression in the placentas of women with pregnancy‐associated VI and the HC women (Figures 1 and 2, Panels B‐C). We studied the expression of both proteins in the different cell types of the placental villi (syncytiotrophoblast, cytotrophoblast and in the foetal capillary) and in the decidual cells of the human placenta. The percentage of syncytiotrophoblast cells that were positively labelled for NOX‐1 protein was significantly higher in the women with pregnancy‐associated VI (79.00 [43.00‐95.00] %) than in the HC group (32.00 [12.00‐45.00] %; *P* < .0001). The percentage of cytotrophoblast cells that expressed NOX‐1 was significantly higher in the women with pregnancy‐associated VI (36.00 [21.00‐56.00] %) than in the HC group (21.50 [11.00‐32.00] %; *P* = .0017). The frequency of the cells in the foetal capillaries of the placental villi that expressed NOX‐1 was significantly higher in the women with pregnancy‐associated VI (31.00 [17.00‐52.00] %) than in the HC group (22.50 [17.00‐31.00] %; *P* = .0435). In the decidual cells of the placenta, we also found a significant increase in the percentage of positive NOX‐1 expression in the women with pregnancy‐associated VI (62.50 [32.00‐94.00] %) with respect to those of the HC group (41.00 [11.00‐71.00] %; *P* < .0001; Figure 1, Panels B‐C).

The NOX‐2 gene expression was also significantly increased in the placentas from women with pregnancy‐associated VI (28.66 [24.18‐39.89] RQ), with respect to that measured in the HC group (26.14 [23.41‐31.27] RQ; *P* = .0109; Figure 2, Panel A). Differences in NOX‐2 protein expression were observed between the VI and HC groups, depending on which cells of the placental villi and the decidual cells were analysed. The frequency of syncytiotrophoblast cells that expressed NOX‐2 was significantly higher in the VI group (49.00 [24.00‐65.00] %) than in HCs (40.50 [18.00‐57.00] %; *P* = .0053). No significant differences in the percentages of positive NOX‐2 expression were observed between women with pregnancy‐associated VI and the HCs in the cells of the cytotrophoblast (23.00 [19.00‐35.00] % vs 16.50 [7.00‐37.00] %, respectively, *P* = .0812) or in the foetal capillaries (38.00 [27.00‐62.00] % vs 32.00 [21.00‐54.00] %, respectively, *P* = 0.0942). The mean percentages of NOX‐2 expression in the decidual cells were 64.50 [37.00‐81.00] % in the VI and 42.00 [23.00‐61.00] % in the HC group, and there was a statistically significant difference in these values (*P* < .0001; Figure 2, Panels B‐C).

### 3.3 Placentas from women with pregnancy‐associated VI show increased iNOS but normal eNOS expression

**Figure 3:**
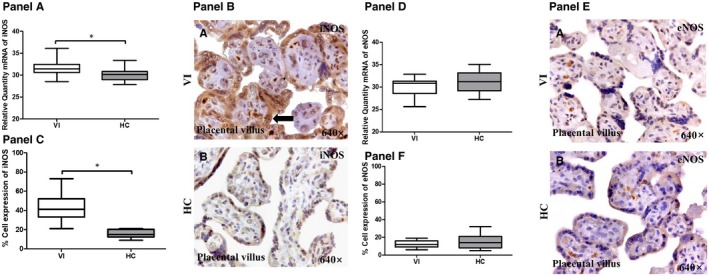
Panel A, iNOS gene expression between pregnant women with lower extremity venous insufficiency (VI) and pregnant women health control (HC), as measured by RT‐qPCR. Panel B, Histological images of iNOS protein expression showing the relevant immunodetection in the placental villi (B) of the placentas from women with VI. Panel C, Quantification of the percentage of cells positive for iNOS in the syncytiotrophoblast cells of the placental villi. Panel D, eNOS gene expression between pregnant women with lower extremity VI and pregnant women health control (HC), as measured by RT‐qPCR. Panel E, Histological images of eNOS protein expression in the placental villi of the placentas from women with VI (A) and of the HC women (B). Panel F, Quantification of the percentage of cells that are positive for eNOS in the syncytiotrophoblast cells of the placental villi. The arrows are the brown coloration indicating the specific protein expression, and 640× is the magnification of histological images. Data shown median and IQR. *P‐value < .05 (Mann‐Whitney *U* test)

We investigated the gene and protein expression of iNOS and eNOS with RT‐qPCR and immunohistochemical techniques in the placentas of women with pregnancy‐associated VI and in the HCs. With RT‐qPCR, we found that the iNOS gene expression in the placentas or women with pregnancy‐associated VI (31.46 [28.51‐36.12] RQ) was significantly higher than in those that were obtained from the HCs (30.14 [27.84‐33.37] RQ; *P* = .0037; Figure 3, Panel A). In contrast, there was no significant difference in the eNOS gene expression in the placentas from both groups of women (VI = 30.86 [25.63‐32.86] RQ vs HC = 31.20 [27.24‐35.08] RQ, *P* = .1845; Figure 3, Panel D).

At protein level, the percentage of syncytiotrophoblast cells that express iNOS was significantly higher in the women with pregnancy‐associated VI (41.00 [21.00‐73.00] %) than in the women of the HC group (15.00 [9.00‐21.00] %; *P* < .0001; Figure 3, Panel C). The eNOS protein expression did not show a significant difference between the study groups in the syncytiotrophoblast cells of the placental villus (VI = 12.00 [6.00‐19.00] % vs 14.00 [5.00‐32.00] %, *P* = .3746; Figure 3, Panels F‐E). Interestingly, there were no significant differences in the expression of iNOS or eNOS small populations of cytotrophoblast, foetal endothelia or decidual capillary cells that were examined in the placentas from both groups of women.

### 3.4 Placentas from women with pregnancy‐associated VI show increased PARP gene and protein expression

**Figure 4:**
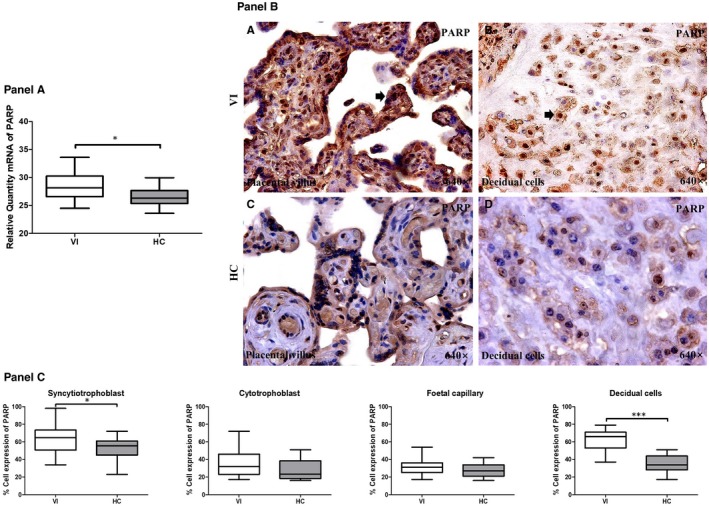
Panel A, PARP gene expression between pregnant women with lower extremity venous insufficiency (VI) and pregnant women health control (HC), as measured by RT‐qPCR. Panel B, Histological images of PARP protein expression showing the relevant immunodetection in the placental villi (C) and the decidual cells (D) in the placentas from women with VI. Panel C, Quantification of the percentages of syncytiotrophoblast, foetal capillary and decidual cells that are positive for PARP. The arrows are the brown coloration indicating the specific protein expression, and 640× is the magnification of histological images. Data shown median and IQR. *P‐value < .05 (Mann‐Whitney *U* test)

We analysed the gene expression of PARP by RT‐qPCR in the placentas of women with pregnancy‐associated VI and in the HCs. Our results showed a significant increase in the level of PARP expression in the placentas from the women with pregnancy‐associated VI (28.13 [24.51‐33.61] RQ) compared to the HCs (26.33 [23.60‐29.95] RQ; *P* = .0071; Figure 4, Panel A).

The protein expression of PARP was examined in the placental villus and decidual cells with IHC techniques. We observed a significant increase in PARP expression in the syncytiotrophoblast cells of the women with pregnancy‐associated VI (65.00 [34.00‐98.00] %) compared to the HC group (55.50 [23.00‐72.00] %; *P* = .0455; Figure 4, Panels B and C). In addition, we observed a significant increase in PARP protein expression in the decidual cells of the women with pregnancy‐associated VI (66.00 [37.00‐79.00] %) compared to the HCs (34.00 [17.00‐51.00] %; *P* < .0001). No significant differences in the percentage of protein expression were observed in the cytotrophoblast and foetal capillary cells from the placentas from both groups of women.

### 3.5 Placentas from women with pregnancy‐associated VI show increased MAPK/ERK gene and protein expression

**Figure 5:**
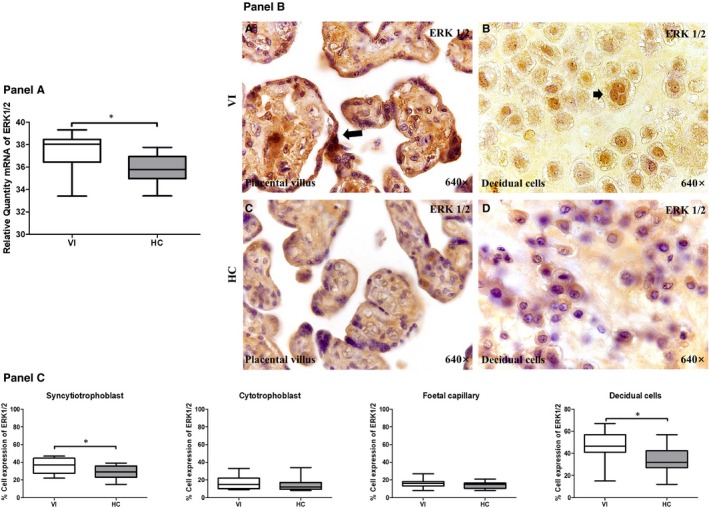
Panel A, ERK 1/2 gene expression between pregnant women with lower extremity venous insufficiency (VI) and pregnant women health control (HC), as measured by RT‐qPCR. Panel B, Histological images of ERK1/2 protein expression showing the relevant immunodetection in the placental villus (C) and decidual cells (D) in the placentas of women with VI. Panel C, Quantification of the percentages of syncytiotrophoblast, cytotrophoblast, foetal capillary and decidual cells that stain positive for ERK 1/2. The arrows are the brown coloration indicating the specific protein expression, and 640× is the magnification of histological images. Data shown median and IQR. *P‐value < .05 (Mann‐Whitney *U* test)

We analysed the gene and protein expression of MAPK, specific ERK 1/2, with the same techniques of study. With RT‐qPCR, we observed that ERK 1/2 gene expression in the placentas of the women with pregnancy‐associated VI (38.04 [33.41‐39.32] RQ) was significantly higher than the HCs (35.80 [33.43‐37.76] RQ; *P* = .0129; Figure 5, Panel A). In the immunohistochemical studies, we observed that the protein expression of ERK 1/2 was significantly higher in syncytiotrophoblast cells in VI placentas than the HCs (VI = 37.00 [22.00‐47.00] % vs HC = 29.00 [15.00‐39.00] %; *P* = .0242). Furthermore, the protein expression of ERK 1/2 was significantly higher in decidual cells in VI placentas than the HCs (VI = 46.50 [15.00‐67.00] % vs HC = 32.00 [12.00‐57.00] %; *P* = .0029; Figure 5, Panels B‐C).

### 3.6 Increased MDA levels in the plasma and placental tissue of women with pregnancy‐associated VI

**Figure 6:**
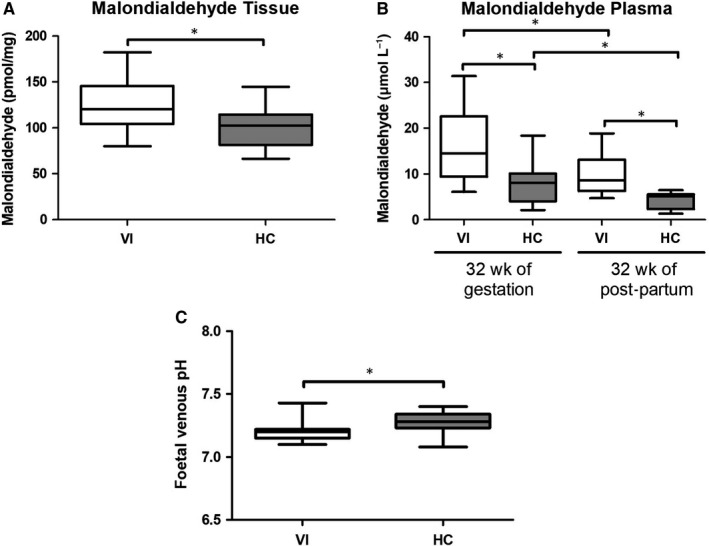
A, Malondialdehyde (MDA) levels in pmol/mg in the placental tissues of pregnant women with lower extremity venous insufficiency (VI) and healthy control (HC) pregnant women. B, Plasma levels of malondialdehyde (MDA) in μmol/L of pregnant women with VI and HCs at 32 wk of gestation and at 32 wk post‐partum. C, Significant decrease of foetal venous pH in mothers with pregnancy‐associated VI. Data shown median and IQR. *P‐value < .05 (Mann‐Whitney *U* test)

The levels of MDA in the placentas from women with pregnancy‐associated VI (120.78 [80‐15‐182.45] pmol/mg) were significantly higher than those found the HC group (102.55 [66.37‐144.67] pmol/mg; *P* = .0084; Figure 6A).

Next, we studied systemic lipid peroxidation, as measured by plasma MDA levels, in both groups of women at the time of inclusion in the study at 32 weeks of pregnancy and at 32 weeks after delivery. We found that the plasma MDA levels in the women with pregnancy‐associated VI were significantly higher than those found in the HC women at both times of the study (VI = 14.55 [6.08‐31.43] μmol/L vs. HC = 8.04 [2.12‐18.39] μmol/L at 32 weeks of pregnancy (*P* = .0227) and VI = 8.64 [4.71‐18.89] μmol/L vs HC = 5.14 [1.36‐6.49] at 32 weeks post‐partum [*P* = .0021]). The plasma MDA levels at pregnancy were significantly higher than those after delivery in both group of women (*P* = .0402 compared VI group, *P* = .0350 compared HC group; Figure 6B).

### 3.7 Foetuses of mothers with pregnancy‐associated VI show a decrease in the umbilical cord venous pH

We investigated the umbilical cord venous pH of foetuses from women with pregnancy‐associated VI and from HC women (Figure 6C). We observed that the foetuses of women with pregnancy‐associated VI had a significant decrease in their venous pH as compared to those from HC women (VI = 7.20 [7.100‐7.43] vs HC = 7.28 [7.08‐7.40], *P* < .0001).

## 4 DISCUSSION

In this paper, we have demonstrated overexpression of enzymes that are critically involved in the induction of oxidative stress and DNA repair in the placental of women with pregnancy‐associated VI. Placentas from women with pregnancy‐associated VI show increased gene and protein expression of NOX1, NOX2, iNOS and PARP. Furthermore, women with pregnancy‐associated VI show increased systemic levels of the oxidative product MDA, which also remained significantly elevated post‐partum.

Pregnancy is a neuroendocrine and mechanical challenge to women that can induce or favour the expression of different disorders, including cardiovascular diseases such as VI.^43^, ^44^ The development of pregnancy‐associated VI has been associated with the development of placental lesions.^9^, ^13^

In this paper, we investigated the pathogenic mechanisms that were involved in the induction of the cellular lesions that were found in the placentas from women with pregnancy‐associated VI. Oxidative stress is a critical mechanism for cellular damage.^19^ We found a specific pattern of overexpression of NOX1 and NOX2 enzymes in the placentas from women with pregnancy‐associated VI. A widespread increased expression of NOX1 in the four different placenta cellular types was observed, as was an enhanced NOX2 expression that was specific to the syncytiotrophoblast and decidual cells in these damaged placentas. Numerous authors have shown how placental oxidative stress has very important role in pregnancy complications.^45^, ^46^, ^47^, ^48^, ^49^ Placentas from women with arterial pregnancy‐associated diseases such as pre‐eclampsia show increased expression of NOX1 in the syncytiotrophoblast and decidual cells.^27^ Furthermore, the increased expression of NOX1 and NOX2 has been described in the syncytiotrophoblast and cytotrophoblast cells in the placentas of women with premature membrane ruptures and intrauterine growth restriction.^50^, ^51^, ^52^ The enhanced cellular expression of these enzymes has been proposed as a marker of metabolic damage to these cells that are pivotal in the physiology of the placenta.^53^, ^54^

Oxidative stress severely impairs cellular metabolism that involves mitochondrial function, and it damages molecules such as DNA.^32^, ^55^, ^56^ The critical metabolism regulator iNOS is expressed in conditions of cellular oxidative stress.^19^, ^52^ We have observed the selective overexpression of iNOS in syncytiotrophoblast cells from the placentas of women with pregnancy‐associated VI. In contrast, normal eNOS expression was found in the four placental cell types that were investigated. Contradictory results have been reported regarding the expression of both types of catalytic enzymes in the placental cells of women with arterial diseases such as hypertension and pre‐eclampsia. Both increased and decreased iNOS and eNOS expression have been described in syncytiotrophoblast cells.^14^, ^57^, ^58^, ^59^ Abnormalities in the expression of iNOS are not merely a marker of oxidative stress but also potential mediators of cellular dysfunction and the abnormal regulation of vascular placental homeostasis.^25^

We also investigated the severity of the oxidative stress that is present in the placentas of women with pregnancy‐associated VI by indirectly analysing the damage to the cellular DNA. This molecular event triggers the response of different protective mechanisms, including the activation of the PARP enzyme.^55^, ^60^ Thus, PARP expression is a biomarker of DNA damage.^32^ Indeed, increased PARP expression has been observed in cytotrophoblast cells suffering from oxidative stress.^49^ Our findings demonstrate an enhanced PARP expression in the syncytiotrophoblast and decidual cells in the placentas of women with pregnancy‐associated VI. However, we cannot ascribe the induction of this DNA repair mechanism solely to oxidative stress, since other mechanisms can also be involved in DNA damage. Recently, it has been shown that a culture medium with a low pH can induce PARP expression in decidual cells.^61^ We found a low pH in the umbilical cord venous blood of women with pregnancy‐associated VI. Thus, the potential involvement of a low local pH in the induction of PARP overexpression in the placental cells may also be proposed. All this can be an explanation of the possible existence of oxidative stress and associated cellular damage, which is related to the acidification of the foetal pH and that could have epigenetic consequences. In these sense, more studies are necessary. In our study, we have not observed other associated complications in foetuses at the time of delivery.

The presence of a cell damage marker is related to the increased expression of ERK in the placentas. In this regard, data on the existence of an ERK deregulation in unfavourable obstetric situations have been reported.^62^, ^63^, ^64^ Recent studies have shown how patients with chronic VI have a greater expression of ERK in their venous wall.^65^ All this makes us think about what was mentioned by other authors that relate the important action of ERK and control gene expression, in particular by regulating transcription.^33^, ^34^ That is why it can have important repercussions in placenta. The increase in the expression of NOX1, NOX2, iNOS, PARP and ERK in the placental villus syncytiotrophoblast in the VI situation is important. They are areas of the placenta that fulfil important functions in maternal and foetal well‐being. In addition, the expression of these molecules in the decidua should be noted, since it is the maternal interface of the embryo and participates in the exchange of gases, nutrients and waste products. The decidua is in relation to the syncytiotrophoblast cells, since the decidua directs and allows a very controlled invasion by the trophoblast membrane.^66^

We were also interested in the analysis of systemic oxidative stress in the placentas of women with pregnancy‐associated VI. ROS are generally unstable and have a very short half‐life; therefore, accurately assessing their levels in the clinical setting is difficult. The established biomarkers of oxidative stress in human samples include the quantification of MDA.^67^, ^68^ We found that the plasma MDA levels were enhanced in women with pregnancy‐associated VI with respect to the HCs. In both populations of women, we observed a significant reduction in the plasma MDA concentrations 32 weeks after delivery, but they remained significantly elevated in the women with pregnancy‐associated VI. Different mechanisms that are not mutually exclusive may be involved in the increased levels of circulating MDA in women with pregnancy‐associated VI at both times of the study (such as venous hypertension that is created by venous insufficiency of the lower extremity, being aggravated by the pressure of the foetus and placenta, and the fact that there is an elevation of oxidative stress markers in the placenta of VI patients). It is known that normal pregnancy is characterized by a low grade of oxidative stress,^69^, ^70^ but we demonstrated markedly higher levels of oxidative stress in the placentas of the women with pregnancy‐associated VI with respect to those of the HCs. Thus, it is possible to suggest that the placentas from these women are a critical source of MDA generation. The significant reduction in the MDA levels 32 weeks after the delivery supports the key role of the placenta in determining the MDA level. However, it is also possible to claim an extraplacental origin of this oxidative stress. Increased local venous wall and systemic oxidative stress have been described in patients with VI.^71^ Recent studies have shown how in patients with VI in chronic situation there is a significant increase in plasma levels of MDA.^72^ The persistence of the increased MDA levels in the women with pregnancy‐associated VI favours a mechanism of oxidative stress that is not solely dependent on the placenta. It is plausible to suggest that women with pregnancy‐associated VI suffer from a more generalized venous disease that remains after their pregnancy.

Future studies with a much larger sample size are necessary to be able to take into account determining factors in foetal well‐being, this point is limiting. Future works should establish the potential clinical relevance of pregnancy‐associated VI and the value of preventive strategies, including the role of angiogenic and proangiogenic factors at a systematic level. Our data support the hypothesis that these women show severe oxidative stress in their placentas as well as a systemic and long period of increased generation of oxidative markers.

## CONFLICTS OF INTEREST

None declared.

## AUTHOR CONTRIBUTIONS

MAO, NGH and JB designed the study and carried out the experimentation. FS, CB, JDL and MAM performed the clinical follow‐up of the patients. MAO, AA and BR created the figures and statistical analyses. MAO, BR and CMV prepared and provided the samples and participated in the immunohistochemistry and gene analysis. MAO, AA, MAM, NGH and JB edited the paper and commented on the interpretation of the results. MAM, NGH and JB participated in study co‐ordination and supervision. All authors read, discussed and approved the final manuscript.

## Data Availability

All relevant data are within the paper and its Supporting Information files.
