# Supplementary material for: Pregnancy‐associated venous insufficiency course with placental and systemic oxidative stress
Source: J Cell Mol Med. 2020 Mar 6;24(7):4157–70. doi: 10.1111/jcmm.15077 (PMC7171392; doi:10.1111/jcmm.15077)
Supplement: Supplementary file 1 [file JCMM-24-4157-s001.docx]

| **GENE** | **SEQUENCE Fwd (5’→3’)** | **SEQUENCE Rev (5’→3’)** | **Temp** | |
| --- | --- | --- | --- | --- |
| **TBP** | TGC ACA GGA GCC AAG AGT GAA | CAC ATC ACA GCT CCC CAC CA | | 60^O^C |
| **eNOS** | AAG AGG AAG GAG TCC AGT AAC ACA GA | ACG AGC AAA GGC GCA GAA | | 60^O^C |
| **iNOS** | CCT TAC GAG GCG AAG AAG GAC AG | CAG TTT GAG AGA GGA GGC TCC G | | 61^O^C |
| **NOX1** | GTT TTA CCG CTC CCA GCA GAA | GGA TGC CAT TCC AGG AGA GAG | | 55^O^C |
| **NOX2** | TCC GCA TCG TTG GGG ACT GGA | CCA AAG GGC CCA TCA ACC GCT | | 60^O^C |
| **PARP** | CCAGGATGAAGAGGCAGTGAAG | TTCTGAAGGTCGATCTCATACTCC | | 58 ^O^C |
| **ERK1/2** | CGC CCC TCC AAA CGG CTC AA | GCA GCG CCT CCC TTG CTA GA | | 62^O^C |
