# Supplementary material for: Pregnancy‐associated venous insufficiency course with placental and systemic oxidative stress
Source: J Cell Mol Med. 2020 Mar 6;24(7):4157–70. doi: 10.1111/jcmm.15077 (PMC7171392; doi:10.1111/jcmm.15077)
Supplement: Supplementary file 2 [file JCMM-24-4157-s002.docx]

| **Antigen** | **Species** | **Clone** | **Dilution** | **Provider** | **Protocol Specifications** |
| --- | --- | --- | --- | --- | --- |
| NOX 1 | Rabbit | Polyclonal | 1:250 | Abcam (ab78016) | 10 mM Sodium citrate pH=6 before incubation with blocking solution |
| NOX 2 | Goat | Polyclonal | 1:500 | Abcam (ab111175) | 100% Triton 0.1% in PBS, 10 minutes, before incubation with blocking solution |
| iNOS | Rabbit | Polyclonal | 1:350 | Abcam (ab95866) | 10 mM Sodium citrate pH=6 before incubation with blocking solution |
| eNOS | Rabbit | Polyclonal | 1:50 | Abcam (ab66127) | EDTA pH=9 before incubation with blocking solution |
| PARP | Mouse | Monoclonal | 1:1000 | Abcam (ab110915) | 10 mM Sodium citrate pH=6 before incubation with blocking solution |
| ERK1/2 | Mouse | Monoclonal | 1:250 | Abcam (ab54230) | 10 mM Sodium citrate pH=6 before incubation with blocking solution |
